# Misinformation and the US Ebola communication crisis: analyzing the veracity and content of social media messages related to a fear-inducing infectious disease outbreak

**DOI:** 10.1186/s12889-020-08697-3

**Published:** 2020-05-07

**Authors:** Tara Kirk Sell, Divya Hosangadi, Marc Trotochaud

**Affiliations:** 1 Johns Hopkins Center for Health Security, Baltimore, USA; 2 Department of Environmental Health and Engineering Johns Hopkins Bloomberg School of Public Health, Baltimore, USA

**Keywords:** Misinformation, Communication, Ebola, Twitter, Social media, Infectious disease

## Abstract

**Background:**

The Ebola communication crisis of 2014 generated widespread fear and attention among Western news media, social media users, and members of the United States (US) public. Health communicators need more information on misinformation and the social media environment during a fear-inducing disease outbreak to improve communication practices. The purpose of this study was to describe the content of Ebola-related tweets with a specific focus on misinformation, political content, health related content, risk framing, and rumors.

**Methods:**

We examined tweets from a random 1% sample of all tweets published September 30th - October 30th, 2014, filtered for English-language tweets mentioning “Ebola” in the content or hashtag, that had at least 1 retweet (*N* = 72,775 tweets). A randomly selected subset of 3639 (5%) tweets were evaluated for inclusion. We analyzed the 3113 tweets that meet inclusion criteria using public health trained human coders to assess tweet characteristics (joke, opinion, discord), veracity (true, false, partially false), political context, risk frame, health context, Ebola specific messages, and rumors. We assessed the proportion of tweets with specific content using descriptive statistics and chi-squared tests.

**Results:**

Of non-joke tweets, 10% of Ebola-related tweets contained false or partially false information. Twenty-five percent were related to politics, 28% contained content that provoked reader response or promoted discord, 42% contained risk elevating messages and 72% were related to health. The most frequent rumor mentioned focused on government conspiracy. When comparing tweets with true information to tweets with misinformation, a greater percentage of tweets with misinformation were political in nature (36% vs 15%) and contained discord-inducing statements (45% vs 10%). Discord-inducing statements and political messages were both significantly more common in tweets containing misinformation compared with those without(*p* < 0.001).

**Conclusions:**

Results highlight the importance of anticipating politicization of disease outbreaks, and the need for policy makers and social media companies to build partnerships and develop response frameworks in advance of an event. While each public health event is different, our findings provide insight into the possible social media environment during a future epidemic and could help optimize potential public health communication strategies.

## Background

The spread of misinformation has become an important global issue. Misinformation contradicts the best expert evidence available at the time and can lead to individual misperception or “factual beliefs that are false or contradict the best available evidence in public domain.” [1, 2] The emergence of new communication platforms and access-enabling technology, such as social media and cell phones, that connect networks of people who often share similar opinions and cultural beliefs, has exacerbated and amplified this problem [3, 4]. Misinformation is not limited to the political realm, where it is widely recognized, but has emerged as an important issue in public health-related messaging and potential barrier to effective disease outbreak preparedness and response [5]. Common examples of health misinformation during public health events or emergencies include false remedies for illness, incorrect information on disease transmission, or allegations that the disease is associated with a government conspiracy [6, 7]. Characterization of health-related misinformation is needed to develop evidence-based risk communication efforts during public health emergencies. In particular, it is necessary to develop more effective strategies to manage misinformation during infectious disease emergencies based on an understanding of typical trends in health-related misinformation.

Although scientific information is generally objective, interpretation and acceptance of such messages can vary based on a person’s identity or personal beliefs. These factors shape information consumption and acceptance in ways that that align with a person’s identity and world view [8–10]. Additionally, when health or science information is framed in the form of “culturally antagonistic memes” that connect such information to divisive social or political issues in provocative ways, risk perception can be altered [9]. Furthermore, the outcome of competition between differing message frames is often dependent on how much each message resonates with an individual’s values [11]. In addition to identity and beliefs, emotion can also play a substantial role in the spread and acceptance of information.

The impact of misinformation during a public health emergency can vary based on its prevalence, content, and persuasive capacity. Moreover, social media may increase its effects, serving as both a source of misinformation and a catalyst for its dissemination [12–14]. Some research has indicated that social media may lead to selective exposure to specific types of content, driving possible “echo chambers” and the formation of homogenous clusters is a primary driver of information diffusion [15]. Other studies assessing this phenomenon note, however, that echo chambers may not be as present depending on how diverse exposure is to different forms of media [16]. False information can spread easily over social media platforms. For example, in Twitter rumor cascades, false news reaches more people than true information [4]. Social media is now one of the key sources in how many people receive their health news and [17, 18], as such, has a key role in the spread of health misinformation.

During infectious disease outbreaks, effective communication is critical for efficient response and recovery efforts. Fear, uncertainty, lack of knowledge, and information seeking behavior among the public may increase opportunities for the propagation of misinformation. The role that misinformation has played fueling vaccine hesitancy has been widely studied [19–21], and outbreaks of vaccine preventable diseases have demonstrated the impacts of misinformation [22]. There has also been increasing focus on politicization and discord-inducing nature of anti-vaccination content, and its connection to political disinformation [23].

The 2013–2016 West Africa Ebola epidemic has provided a unique avenue through which to assess this issue, as misinformation related to Ebola was common. Despite very low case counts within the United States (US), widespread fear and media attention contributed to dissemination of US Ebola-related misinformation [7]. Coverage from traditional media sources was dispersed on social media, where it joined a mixture of factual and false information [7, 24–26]. News and social media content during the Ebola epidemic has been explored both within West African [25, 27, 28], American contexts [7, 24], and European contexts [29]. Research focusing on Ebola-related misinformation in the West African context identified common rumors including false treatments; misinformation about the intentions and motives of healthcare workers treating Ebola patients; and rumors about the Ebola epidemic being a hoax [27, 28]. Such rumors may have impeded the ability for public health responders to communicate to communities about effective prevention and control methods. Research on Ebola-related misinformation in a predominantly Western context found that rumors such as the existence of “Ebola zombies”, misperceptions about the disease transmission, or inaccurate information about experimental Ebola vaccines was common, potentially leading to fear, uncertainty, and confusion amongst the public [7].

Such literature has provided a high-level characterization of the general types of correct and incorrect Ebola-related information circulating on social media. Further research is needed, however, to understand specific characterizations and content of correct, false, and partially false information, and possible trends in the existence political or discord-inducing messages. The goal of this research is to address such gaps in the literature by providing an in-depth analysis of twitter content, particularly misinformation, in the context of an outbreak that generated high levels of fear and attention amongst the US population. Specifically, we aim to describe the content of Ebola-related tweets with a specific focus on the characteristics of misinformation, political content, health related content, risk framing, and rumors. While each public health event is different, this research may provide insight into the possible social media environment during a future epidemic.

## Methods

### Study design and setting

We conducted a quantitative content analysis of Ebola-related tweets published between September 30th and October 30th, 2014. On September 30th, 2014 the first domestic diagnosis of Ebola occurred in the US. Studies of news media, tweet, and google search volume have identified the one-month period following this event as a time of high interest in Ebola amongst the US population and was thus the most applicable time period for the study goal of analyzing miscommunication during high levels of fear for the US population [24, 30].

### Sample selection

We examined tweets from a random 1% sample of all tweets published during the time period of interest, using the Twitter Application Programming Interface (API) to record the official 1% sample stream. This sample was then filtered for tweets that mentioned “Ebola” in the tweets’ content or hashtag, were English language, and had a minimum of 1 retweet (*N* = 72,775 tweets). The latter requirement was used to ensure at least some social interaction with the tweet. All tweets in the dataset represented original published content as determined by the unique Tweet identifier assigned by the Twitter API. A randomly selected subset of 3639 (5%) tweets, determined to be the largest dataset the research team could code with available resources, was then analyzed using public health-trained human coders. Tweets were excluded if the content was unrelated to Ebola.

### Data collection

An initial coding instrument was developed based on a literature review of misinformation and Ebola-related research. Prior to coding, 277 tweets, not included in the analytical sample, were randomly selected to pilot and refine the coding instrument. Piloting was conducted until all three authors were in agreement on coding methodology and interpretation of coding instrument. The final coding instrument included dichotomous yes/no codes assessing tweet characteristics (e.g. joke, opinion, discord), veracity (true, false, partially false), political context, risk frame [31], health context, Ebola specific messages [30, 32], rumors [7], and 23 hashtags previously identified in literature as potentially suspicious or associated with misinformation (Additional File 1) [25]. Two authors (DH and MT) coded tweets using a Google survey form. The extensive piloting process indicated a need for collaborative review of some tweets that were challenging to code. As a result, a system in which three authors (DH, MT, and TKS) reviewed those tweets in question was established and continued throughout the coding process. Two authors (DH and MT) assessed interrater reliability for each dichotomous yes/no item by each coding the same random sample of 200 tweets. All but one item had kappa values of 0.79 of higher and therefore met conventional standards for adequate reliability [33]. The remaining item – statement in support of government - did not have a satisfactory Kappa value but remained in the coding instrument because it was very rare and showed 99.5% agreement.

### Data analysis

We assessed the specific content of tweets using descriptive statistics to determine the frequency of each coding item. Then, we used Pearson’s chi-square tests to compare specific content (eg risk elevated messaging, discord, political statements) in tweets with misinformation compared to those without misinformation. The dataset was also stratified by the number of retweets. For strata of at least 2, over 10, or over 100, the percentages of tweets containing misinformation, discord-inducing statements, or statements opposed to government were determined and compared. Statistical analyses were conducted using Stata 15 [34].

## Results

Of the 3639 coded tweets, 526 (14%) were excluded either for irrelevant language, foreign language, or because coders considered them to be peripheral to the Ebola outbreak (e.g. focused on sporting events, Halloween costumes, or other unrelated topics). The remainder of the analysis is focused on the final set of 3113 tweets.

Tweets fell into three broad categories: health information, political statements, and jokes. Health information statements (60%) generally featured information about the disease, its potential spread, and transmission (e.g. “I’m sick of y’all and the ignorance about Ebola. Ebola is harder to catch than the common cold and is spread by blood and bodily fluids”). Political statements (21%) focused on ongoing political issues or figures (e.g. “Why does our President want to raise our electric bills, ruin our health insurance and FLOOD our country w Ebola &amp; illegal immigrants? #tcot”).^1^ Jokes (21%) included text (e.g. “Bad news: Ebola in Dallas. Good news: Tony Romo won’t let anyone catch it. #cowboys #ebola”), gifs, and photos. There was overlap within these three categories, and in total 88% of all tweets fell in to at least one of the three. The largest overlap (10%) was between health information and political sentiments. To better characterize circulating messages about Ebola, additional analyses were performed after joke tweets were removed. Without jokes, 72% of tweets were related to health and 25% were related to politics. (Table 1).

**Table 1:** Characteristics of Tweets about Ebola

	Full Data Set	Data Set Without Jokes
**Descriptive Qualities**	Frequency (N)	Frequency (N)
Tweet Interpreted as a joke	21% (653)	N/A
Tweet Contains News Headline	7% (204)	8% (204)
Tweet Shares True Information	31% (953)	38% (941)
Tweet Shares Half-true Information/ Misrepresents the truth	4% (128)	5% (120)
Tweet Shares False Information	4% (134)	5% (125)
Unable to ascertain the Truth in Tweet	12% (365)	15% (363)
Tweet Shares an Opinion	42% (1318)	52% (1286)
Tweet Designed to Promote Discord/ Evoke a Response	22% (696)	28% (689)
**Political Content**		
Content of Tweet Political in Nature	21% (644)	25% (625)
Sentiments in Support of Gov	< 1% (11)	< 1% (11)
Sentiments in Opposition of Gov	11% (352)	14% (343)
**Risk Frames**		
Tweet Contains Risk Elevating Message	35% (1077)	42% (1045)
Tweet Contains Risk Minimizing Message	12% (365)	14% (355)
**Ebola Specific Content**		
Tweet Shares Sentiments Related to Health	60% (1863)	72% (1768)
Tweet Mentions Medical Counter Measures	2% (71)	3% (64)
Tweet Mentions Fatal Nature of Ebola	7% (213)	8% (200)
Tweet Mentions the Spread of the Outbreak	30% (929)	35% (854)
Tweet Mentions the Reduction of the Outbreak	4% (109)	4% (107)
Tweet Mentions Travel Ban/Closing Border	2% (70)	3% (70)
Tweet Mentioned Quarantine/Isolation	3% (104)	4% (102)
Tweet Mentioned Screen/ Fever Check at Airports	1% (31)	1% (30)
Tweet Mentioned Public Health Monitoring	1% (38)	2% (38)
Percentage of Tweets Mentioning at Least One of Prior Categories	44% (1365)	61% (1267)
**Ebola Rumors**		
Tweets that Mention a Rumor	7% (227)	8% (205)
Tweets that Refute a Rumor	1% (45)	2% (43)
**Number of Tweets**	3113	2460

Table 1**:** The full dataset (*n* = 3113 tweets) contained all included tweets related to Ebola. The dataset without jokes (*n* = 2460) excluded all tweets coded as jokes to further focus analysis on Ebola-specific tweet content.

### Misinformation and opinion

**Fig. 1:**
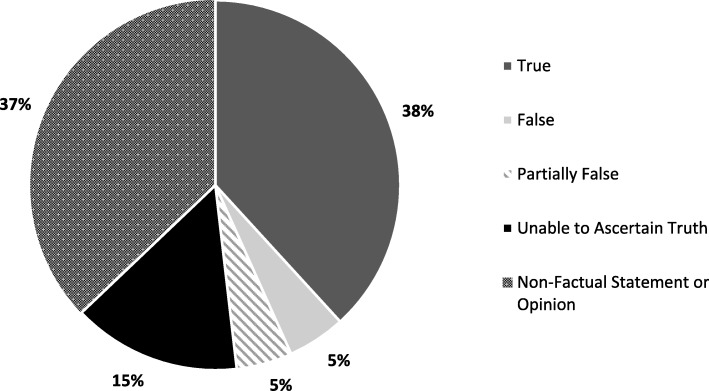
**Percent Distribution of Non-Joke Tweets Containing Factual Statements.** Statements containing facts were determined to be true, false, partially false, or of unknown truth. A total of 37% of non-joke tweets contained statements that did not contain factual statements for which truth could be assessed

Of non-joke tweets, 10% of Ebola-related tweets contained false (5%) or partially false/misinterpreted (5%) information (Fig. 1). Tweets with false information often focused on debunked rumors. For example, “Renown #NSA #Whistleblower: #Ebola Could Be Staged Event To Pillage Africa’s Natural Resources | EPIC @Infowars #News.”^2^ Half-true tweets or those misinterpreting the truth generally included true information but also suggested something that was not true. For example, the following tweet correctly notes that a patient was being tested for Ebola but suggests that there was an actual case: “THERE IS AN EBOLA PATIENT IN A FAIRFAX COUNTY HOSPITAL I’M GOING TO CANADA.” Coders determined that 38% of Ebola-related tweets contained true information. The majority (52%) of non-joke tweets contained one or more opinion statements. Of the tweets containing opinion statements, 73% contained only opinions without any factual statements (including true, partially true, or false information) to support claims.

### Political and discord-promoting content

Excluding joke tweets, 25% of tweets were related to politics while 28% contained content that provoked reader response or promoted discord. Discord-promoting tweets were those that seemed designed to generate a response from and conflict with other Twitter users. Among the tweets containing one of the hashtags of interest, 83% contained the political #tcot (Top Conservatives On Twitter) and about 3% contained the #p2 (Progressives 2.0) hashtags. A small number (*n* = 5) included in the same tweet both conservative and liberal hashtags, enabling opposing political spectrums to the tweet and engage with each other.

Examples of the use of political hashtags include:

- “We have to fight Obama and stop him from bringing Ebola patients from Africa to US”. #BanFlights #NoEbolaPatients #tcot #tiot^3^ #WAARMedia
- “Goodnight. Remember: The GOP”^4^ healthcare plan will kill you long before #Ebola will: #p2
- “#GOP is worried about #Ebola getting over the border... Mr. Duncan Flew in Legally [profanity]; he didn’t sneak in from Mexico #tcot #p2 #bbc”^5^

Additionally, political tweets often intersected Ebola-related issues with other social or political issues debated in American society, indicating that the political nature of Ebola-related discourse extended beyond topics directly related to the epidemic itself, such as racial inequity, government spending, and global warming.

For example:

- “Ebola Victim’s Nephew Speaks: Thomas Eric Duncan Died Because He Was Black And Uninsured http://t.co/7am3tc3VFJ”
- “Dear @SpeakerBoehner, If we’re too broke for Food Stamps, veterans benefits or an Ebola vaccine, why do we still need Big Oil subsidies?”
- “Unlike Global Warming, Ebola is real... But look at which one the left laughs off. Anti-human, y’all. Who they are.”

**Fig. 2:**
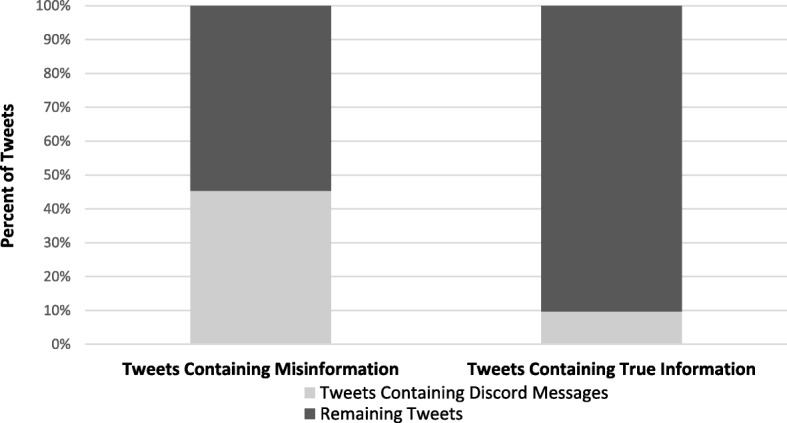
**Percent Distribution of Tweets Containing Discord-Promoting Statements Among Tweets with Misinformation or True Information.** Light gray shading indicates the percentage of tweets with misinformation or true information that also contained discord-promoting statements (*n* = 111 of 245 misinformation tweets) or true information (*n* = 91 of 941 true tweets)

The proportion of non-joke tweets with discord-inducing statements was significantly higher among tweets with misinformation compared to those without misinformation (eg true tweets, opinion tweets, tweets for which truth could not be ascertained) (45% vs 26%, *χ*^2^ = 40.38; *df* = 1; *p* < 0.001) (Table 2). Similarly, the proportion of tweets with political content was significantly higher among tweets with misinformation compared to those without misinformation (36% vs 24%, *χ*^2^ = 15.87; *df* = 1; p < 0.001) (Table 2). Examining tweets with misinformation to the subset of tweets with true information specifically, a greater percentage of tweets with misinformation were political in nature (36% vs 15%) and contained discord-inducing statements (45% vs 10%) (Fig. 2). Additionally, 59% of tweets that were political in nature also contained discord-inducing statements. Over half (55%) of political statements were statements critical of government or of a political figure. About 2% of political tweets expressed statements of support for government, of which, half also contained critical statements. Remaining political tweets were neutral statements.

**Table 2:** Comparing discord-promoting, political, risk-increasing characteristics among tweets with and without misinformation

Tweet Characteristic	Tweets Containing Misinformation	Tweets Without Misinformation	χ^**2**^ Value	***P*** value	Degrees of freedom
Promotes discord	45%	26%	40.38	< 0.001	1
Political	36%	24%	15.87	< 0.001	1
Risk Increasing	76%	39%	127.58	< 0.001	1

Table 2**:** Chi-squared testing found that content promoting social discord, political statements, and risk increasing messaging were all more common in tweets containing misinformation compared to tweets without misinformation. Percentages of tweets with or without misinformation that contain the tweet characteristic of interest are provided, as well as the χ^2^ value, *p*-value, and degrees of freedom.

### Risk perception frames and specific Ebola topics

Although messages with factors that could increase or decrease the perception of risk were not always present in non-joke tweets, when evaluating messages using Slovic’s risk perception framework [31], 42% of non-joke tweets contained features that could contribute to increased perception of risk. Risk elevating messages were significantly more frequent in tweets containing misinformation compared to tweets without misinformation (76% vs 39%, *χ*^2^ = 127.58; *df* = 1; *p* < 0.001) (Table 2). The most frequent Ebola-specific message type was related to the spread of the outbreak (35%), defined as descriptions of increases in case numbers in West African countries as well as new cases in other parts of the world. The second-most common message type was related to the fatal nature of Ebola (8%). Both of these Ebola-specific message types were generally coded as risk-elevating.

### Rumors

Adapted from prior literature and based on piloting [7], we developed a list of 11 key rumors we believed to be the most common circulating during the Ebola epidemic, and marked additional rumors as “other”. Overall, 8% of tweets mentioned an Ebola related-rumor and 2% refuted untrue rumors. Most often (*N* = 61), these rumors focused on the idea that the Ebola outbreak was a conspiracy created by political figures, the news media, or pharmaceutical companies. The next most frequently mentioned rumor (*N* = 46) focused on airborne transmission. Whereas the rumor that the outbreak was a conspiracy was refuted only 7% of the time (*n* = 4), more than half (58%, *n* = 26) of tweets about airborne transmission of Ebola refuted the rumor. Given the prevalence of certain rumors in jokes, the analysis of rumors was inclusive of joke tweets.

### Message characteristics among highly Retweeted content

The median number of retweets for included tweets was 6 per tweet, and the interquartile range was 23 (2 to 25) retweets. The number of retweets was strongly skewed; the tweets in the top 25% percentile ranged from 25 retweets to 14,674, whereas the bottom 25% consisted of tweets retweeted 1 to 2 times. In total, there were 1868 non-joke tweets with at least 2 retweets, 934 non-joke tweets with over 10 retweets, and 229 non-joke tweets with over 100 retweets. Overall, the percentage of tweets with key characteristics, including misinformation and discord was only slightly less in tweets that were more frequently retweeted. Among non-joke tweets, nearly 7% of those with over 100 retweets (*n* = 15) contained misinformation, compared to 9% (*n* = 175) of tweets that contained at least 2 retweets. Additionally, about 23% (*n* = 53) of tweets with over 100 retweets contained discord-inducing messages, compared to 25% (*n* = 230) of tweets with over 10 retweets and 28% (*n* = 521) of tweets with at least 2 retweets. Similarly, the frequency of tweets that were political in nature and were opposed to government decreased only slightly and only after exceeding 100 retweets (56% among tweets with over 1 (*n* = 278) and 10 retweets (*n* = 135); 50% among tweets with at least 100 retweets (*n* = 24)).

## Discussion

### Misinformation and the mixture of true and false information

Results demonstrate that although misinformation was not found in the majority of analyzed tweets, it was still present at notable levels. Our results, that 5% of Ebola related tweets were false and another 5% contained half-true or misinterpreted information in the time period from September 30–October 30, 2014, highlight a potential increase in misinformation from the 2% reported from Twitter and Sina Weibo content in August 2014 [6]. Although differences in sampling and coding methods may limit the comparability of these findings, the increase in misinformation in the later time period may have resulted from the increase attention on the Ebola outbreak after the disease was diagnosed in the US [25].

Considering the networked nature of Twitter, some users were likely to have received a much greater percentage of Tweets containing misinformation. This is particularly important when considering that message frames that are viewed more frequently often have greater influence on public opinion [35]. An analysis of Ebola twitter cascades shows that users receiving misinformation were likely immediate followers of (or the followers of followers of) those that broadcasted misinformation, highlighting the importance of influential Twitter users both the spread and potential control of misinformation [36]. Furthermore, within misinformation containing tweets, the high frequency of partially true or misinterpreted information may also present challenges for public health communicators, particularly if the tweet author cites legitimate sources to support his or her claims. Additionally, more frequent framing of true information in ways that mischaracterize it may solidify this misinterpretation in the minds of the public [37]. Interestingly, slightly more than half of the tweets mentioning Ebola as an airborne disease also refuted it, highlighting the potential of crowd-sourced correction of misinformation [38].

### Intersection of misinformation, political content, and discord

Findings also indicate greater than expected politicization of a seemingly neutral international health emergency. Past research indicates that the types of social media content can vary over the course of an outbreak and political framing of social media messages in past outbreaks appeared more frequently during the “maintenance” stage of outbreak response, which occurs during peak activity of response efforts prior to the resolution of an outbreak [39]. Ebola response activities within the US and the time period of interest in this study corresponded with the lead up to US midterm elections (which occurred Nov 4, 2014). This temporal overlap may have contributed to the political content found in many tweets, particularly with respect to discourse on travel bans and quarantine procedures. Furthermore, the politicization of this health issue extended beyond discussion about the disease itself, and often touched on other contentious political issues in the US, such as immigration, general health care policy, and government spending. These findings support previous research tying the acceptance of information and perceived risk perception with whether such information would align with a person’s political identity [8]. The intersection of health misinformation with political discourse may have also contributed to Ebola response efforts being viewed as partisan topics. As other researchers have noted [8, 10], when a statement is associated with a particular conservative or liberal viewpoint, readers that align with that political ideology may be less likely to critically assess the truth to the statement, which posed a challenge for promoting evidence-based policies in public health response.

Not surprisingly, nearly all political tweets that provided an opinion about government were critical rather than supportive. Additionally, a substantial proportion of these tweets were framed in ways that could promote discord or bait readers into responding. Similar trends have been observed by other researchers assessing vaccine misinformation on social media [23]. Published literature on misinformation during the H1N1 pandemic have also shown that misinformation circulating during pandemics often elicits lack of trust in government or traditional institutions [40]. Notably, the prevalence of tweet characteristics of concern, including the presence of misinformation, discord-inducing statements, and political messaging, remained relatively constant even among highly retweeted content. Given the relationship between political content and misinformation, another potential approach to ensure that accurate information is included in political discourse is to provide political leaders with frequently updated disease information fact sheets. Federal health officials, for example the Health and Human Services Assistant Secretary for Public Affairs and Assistant Secretary for Legislation, can provide frequent updates and concise information to policymakers and political staff members to ensure that science-based health information is easily available. Furthermore, practitioners, policymakers, and the national security community should be aware of the potential usage of public health emergencies to create discord amongst the public during public health emergencies and strive to reduce these effects. Experience with outbreak emergencies has shown the deleterious effects of misinformation and diminishing trust [5].

### Elevating perception of risk – fuel for public fears

The identification and transmission of Ebola in the US generated high levels of fear and concern amongst the public. Results showing high frequency of risk perception elevating tweets align with previously published literature on social media content during the Ebola epidemic [7, 25]. The high proportion of these messages, paired with inflammatory political statements and misinformation demonstrate how public health response efforts and public health messaging can be interpreted or distorted in ways that promote political and social discord. Furthermore, driven by public interest, mass media may sensationalize or exaggerate the risk of disease in a population, which could increase risk perception and risk-elevating messages on social media [24]. Although appropriate levels of risk perception in situations that warrant public attention, investment in protective actions, and policy intervention is necessary, messages that elevate public perception of risk situations that do not warrant high levels of public concern are problematic. Disease outbreaks may naturally lead to messages that could increase perception of risk and public health communicators must work with transparency and empathy for public fears [41].

### Potential approaches to misinformation during health emergencies

Developing a strategy to combat misinformation during an epidemic, particularly one that induces increased levels of fear amongst the public, is challenging, and effectively addressing misinformation will likely involve multifaceted approaches, such as developing compelling public health awareness campaigns, engaging in media literacy efforts, and monitoring of circulating content to develop proactive responses to emerging communication issues. Public private partnerships between social media companies and public health agencies to promote public health messages is also an important component in combating misinformation, as observed in the partnership between the World Health Organization and social media companies to combat the novel coronavirus disease 2019 (COVID-19) misinformation [42, 43]. As governments and global health organizations become increasingly aware of the dangers of misinformation in both health and political sectors, more research will need to be done to ensure that strategies to reduce misinformation are effective and do not infringe of the spread of true information or the ability of members of the public to speak freely. Research assessing the effects of correcting health misinformation has shown varied results. While some studies have indicated that correction of certain misinformation by trusted sources can help reduce misperceptions, others indicate correction can sometimes have limited impact or even contribute to further misperceptions in certain circumstances due to factors such as the perceived trustworthiness of the source and whether corrective information conflicts with the social or political identity of the individual [44, 45]. Furthermore, countering of messages will require clear understanding of the underlying drivers and motivations among stakeholders for promoting false information.

### Limitations

This research is subject to several limitations. Tweets are short and provide limited content to frame coding. The coding process involved human interpretation of sometimes confusing material and may have introduced bias or error. Sarcasm and satire were occasionally difficult to interpret. However, this was mitigated through continual consensus seeking efforts with the team throughout the coding process for tweets considered particularly confusing or challenging. Frequent discussions prevented coders from deviating from agreed upon code definitions over the course of the coding process. Although interrater agreement for coders was high, double coding was conducted on a limited 5% of the sample. Another potential limitation is our decision not to limit the study sample to US geotagged tweets due to concerns about potential bias this may have introduced– that users who allowed geotagging may have been different from other types of Twitter users. Instead, the sample was limited to English language tweets during a time period of heightened attention to Ebola in the US. As a result, not all tweets may have originated in the US. Additionally, codes to assess political nature of tweets were not specific to a particular ideology or political party. Therefore, we were not able to systematically quantify the political leanings of different tweets. Furthermore, tweets were automatically and randomly selected as part of the 1% sample of tweets published in our timeframe of interest. As a result, tweets with low retweet counts may have been published just prior to data capture, potentially showing fewer retweets than they eventually received. Finally, the time period for our study was limited to the period of most intense interest amongst the US population but may have been too short to identify general trends in Ebola-related social media posts.

## Conclusion

Misinformation and its impact on public beliefs is a growing problem in the public health field and will affect all types of disease events - from localized outbreaks of measles [22], to regional outbreaks of Ebola [5], and even potentially significant pandemics [46]. This phenomenon may be an even greater challenge in outbreaks that induce high levels of fear. Results demonstrated that most analyzed tweets were risk elevating, and that misinformation was not a dominant feature but notably present. Findings also highlight the importance of anticipating politicization of disease outbreaks, and the presence of provocative, discord-inducing messages in response to public health measures. Policy makers, public health practitioners, and social media companies will need to work together to develop communication strategies and response frameworks in advance of outbreaks to mitigate misinformation and the resulting public health impacts.

Additional information.

Tara Kirk Sell and Divya Hosangadi are joint first authors.

## Supplementary information

**Additional file 1.** Analytical Instrument. Description of Data: Coding instrument used to conduct content analysis of included tweets.

**Additional file 2.** Tweet IDs. Description of Data: Tweet IDs of included and excluded tweets

## Data Availability

The dataset of tweet IDs used in this study is available as Additional File 2.

## Abbreviations

US: United States
API: Application Programming Interface
#tcot: A hashtag that stands for Top Conservatives on Twitter
NSA: United States National Security Agency
#p2: Progressives 2.0
#tiot: A hashtag that stands for Top Independents on Twitter
GOP: Grand Old Party, a term used to describe the US Republican Party.
#bbc: A hashtag that stands for British Broadcasting Corporation, a British news organization
COVID-19: Coronavirus disease 2019

## Footnotes

1 #tcot is a hashtag that stands for Top Conservatives on Twitter

2 NSA is an abbreviation for the United States National Security Agency

3 #tiot is a hashtag that stands for Top Independents on Twitter

4 GOP stands for Grand Old Party, a term used to describe the US Republican Party

5 #bbc is a hashtag that stands for British Broadcasting Corporation, a British news organization

## References

[1] Vraga EK, Bode L (2020). Defining misinformation and understanding its bounded nature: using expertise and evidence for describing misinformation. Polit Commun.

[2] Flynn DJ, Nyhan B, Reifler J (2017). The nature and origins of misperceptions: understanding false and unsupported beliefs about politics. Polit Psychol.

[3] Lazer DMJ, Baum MA, Benkler Y (2018). The science of fake news. Science.

[4] Vosoughi S, Roy D, Aral S. The spread of true and false news online 2018:7. 10.1126/science.aap9559 29590045

[5] Vinck P, Pham PN, Bindu KK, Bedford J, Nilles EJ (2019). Institutional trust and misinformation in the response to the 2018–19 Ebola outbreak in north Kivu, DR Congo: a population-based survey. Lancet Infect Dis.

[6] Fung IC, Fu KW, Chan CH, Chan BS, Cheung CN, Abraham T, Tse ZT (2016). Social media's initial reaction to information and misinformation on Ebola, august 2014: facts and rumors. Public Health Rep.

[7] Jin F, Wang W, Zhao L, Dougherty ER, Cao Y, Lu CT, Ramakrishnan N (2014). Misinformation propagation in the age of twitter. IEEE Computer.

[8] Kahan DM. Misconceptions, misinformation, and the logic of identity-protective cognition. SSRN Electron J. 2017. 10.2139/ssrn.2973067.

[9] Kahan DM, Jamieson KH, Landrum A, Winneg K (2017). Culturally antagonistic memes and the Zika virus: an experimental test. J Risk Res.

[10] Kahan DM, Peters E, Wittlin M (2012). The polarizing impact of science literacy and numeracy on perceived climate change risks. Nat Clim Chang.

[11] Chong D, Druckman JN (2010). Dynamic public opinion: communication effects over time. Am Pol Sci Rev.

[12] Friggeri A, Adamic L, Ecles D, Cheng J. Rumor Cascades. Assoc Adv Artif Intell. 2014. https://www.aaai.org/ocs/index.php/ICWSM/ICWSM14/paper/viewFile/8122/8110.

[13] Shin J, Jian L, Driscoll K, Bar F (2018). The diffusion of misinformation on social media: temporal pattern, message, and source. Comput Hum Behav.

[14] Scanfeld D, Scanfeld V, Larson EL (2010). Dissemination of health information through social networks: twitter and antibiotics. Am J Infect Control.

[15] Del Vicario M, Bessi A, Zollo F, Petroni F, Scala A, Caldarelli G, Stanley HE, Quattrociocchi W (2016). The spreading of misinformation online. Proc Natl Acad Sci.

[16] Dubois E, Blank G (2018). The echo chamber is overstated: the moderating effect of political interest and diverse media. Inf Commun Soc.

[17] Sarasohn-Kahn J. The wisdom of patients: health care meets online social media. http://www.chcf.org/topics/chronicdisease/index.cfm?itemID=133631. Accessed 26 Oct 2019.

[18] Vance K, Howe W, Dellavalle RP (2009). Social internet sites as a source of public health information. Dermatol Clin.

[19] Kata A (2012). Anti-vaccine activists, web 2.0, and the postmodern paradigm – an overview of tactics and tropes used online by the anti-vaccination movement. Vaccine.

[20] Sarathchandra D. A survey instrument for measuring vaccine acceptance. Prev Med. 2018;7. 10.1016/j.ypmed.2018.01.006 29337069

[21] Gunaratne K, Coomes EA, Haghbayan H (2019). Temporal trends in anti-vaccine discourse on twitter. Vaccine.

[22] Pager T ‘Monkey, Rat and Pig DNA’: How Misinformation Is Driving the Measles Outbreak Among Ultra-Orthodox Jews. *The New York Times*. https://www.nytimes.com/2019/04/09/nyregion/jews-measles-vaccination.html. Published April 9, 2019. Accessed October 11, 2019.

[23] Broniatowski DA, Jamison AM, Qi S (2018). Weaponized health communication: twitter bots and Russian trolls amplify the vaccine debate. Am J Public Health.

[24] Towers S, Afzal S, Bernal G, et al. Mass media and the contagion of fear: the case of Ebola in America. PLoS One. 2015;13. 10.1371/journal.pone.0129179 PMC4465830 26067433

[25] Kalyanam J, Velupillai S. Facts and Fabrications about Ebola: A Twitter Based Study. arXiv preprint arXiv:1508.02079. 2015.

[26] Househ M (2016). Communicating Ebola through social media and electronic news media outlets: a cross-sectional study. Health Inform J.

[27] Oyeyemi SO, Gabarron E, Wynn R. Ebola, Twitter, and misinformation: a dangerous combination? BMJ. 2014;349(oct14 5):g6178-g6178. doi:10.1136/bmj.g6178. 10.1136/bmj.g6178 25315514

[28] Cheung EY (2015). An outbreak of fear, rumours and stigma: psychosocial support for the Ebola virus disease outbreak in West Africa. Intervention.

[29] Van Lent LG, Sungur H, Kunneman FA, Van De Velde B, Das E (2017). Too far to care? Measuring public attention and fear for Ebola using twitter. J Med Internet Res.

[30] Sell TK, Boddie C, McGinty EE (2017). Media messages and perception of risk for Ebola virus infection. United States Emerg Infect Dis.

[31] Slovic P (1987). Perception of risk. Science.

[32] Sell TK, Boddie C, McGinty EE (2016). News media coverage of U.S. Ebola policies: implications for communication during future infectious disease threats. Prev Med.

[33] Landis JR, Koch GG (1977). The measurement of observer agreement for categorical data. Biometrics.

[34] StataCorp (2017). Stata statistical software: release 15.

[35] Chong D, Druckman JN (2007). Framing theory. Annu Rev Polit Sci.

[36] Liang H, Fung IC, Tse ZT, Yin J, Chan CH, Pechta LE, Smith BJ, Marquez-Lameda RD, Meltzer MI, Lubell KM, Fu KW (2019). How did Ebola information spread on twitter: broadcasting or viral spreading?. BMC Public Health.

[37] Chong D, Druckman JN (2007). A theory of framing and opinion formation in competitive elite environments. J Commun.

[38] Bode L, Vraga EK (2018). See something, say something: correction of global health misinformation on social media. Health Commun.

[39] Tang L, Bie B, Zhi D (2018). Tweeting about measles during an outbreak: a semantic network approach to the framing of emerging infectious diseases. Am J Infect Control.

[40] Chew C, Eysenbach G. Pandemics in the Age of Twitter: Content Analysis of Tweets during the 2009 H1N1 Outbreak. PLoS One. 2010;5(11). 10.1371/journal.pone.0014118. 10.1371/journal.pone.0014118 PMC2993925 21124761

[41] US Centers for Disease Control and Prevention. Crisis and Emergency Risk Communication (CERC) manual. https://emergency.cdc.gov/cerc/manual/index.asp. Accessed October 21, 2019.

[42] Mark Zuckerberg [Internet]. [cited 2020 Mar 11]. Available from: https://www.facebook.com/zuck/posts/10111615249124441.

[43] Zarocostas J (2020). How to fight an infodemic. Lancet.

[44] Carey JM, Chi V, Flynn DJ, Nyhan B, Zeitzoff T. The effects of corrective information about disease epidemics and outbreaks: Evidence from Zika and yellow fever in Brazil. Science Advances. 2020 Jan 1;6(5):eaaw7449. 10.1126/sciadv.aaw7449 PMC6989147 32064329

[45] Vraga EK, Bode L (2017). Using expert sources to correct health misinformation in social media. Sci Commun.

[46] Schoch-Spana M, Cicero A, Adalja A (2017). Global catastrophic biological risks: toward a working definition. Health Secur.

