# Supplementary material for: Misinformation and the US Ebola communication crisis: analyzing the veracity and content of social media messages related to a fear-inducing infectious disease outbreak
Source: BMC Public Health. 2020 May 7;20:550. doi: 10.1186/s12889-020-08697-3 (PMC7202904; doi:10.1186/s12889-020-08697-3)
Supplement: Supplementary file 1 — Additional file 1. Analytical Instrument. Description of Data: Coding instrument used to conduct content analysis of included tweets. [file 12889_2020_8697_MOESM1_ESM.docx]

| Name of Code | Definition & Example | | | Measure |
| --- | --- | --- | --- | --- |
| Tweet Qualifiers |  | | |  |
| Tweet ID | A tweet ID is a unique Twitter code assigned to each tweet gathered through the Twitter API.  Example: 517502616541351000 | | | From Twitter API |
| Code number | A code number was assigned for each tweet included in our data set. | | | Counts |
| Coder ID | Each Coder annotated their codes with a distinct coder ID. | | | Internal ID |
| Retweet Count | Our data set was composed of unique tweets with varying numbers of retweets (threshold, tweets with one or more retweet). This value was provided in the data from twitter’s API | | | From Twitter API |
| User Screen Name | Each tweet in the API set came with the public screen name associated with the sender. These names were recorded | | | From Twitter API |
| Tweet Excluded | Certain tweets were excluded for our sample for irrelevance to the outbreak, the peripheral nature of Ebola in the Tweet, or if they fell outside of our inclusion criteria (e.g. some non-English language tweets made it into the set). Throughout coding, exclusions were talked about amongst the three coders.  Example: “COMING OCTOBER 21 2014. #Ebola #Techno #EDM #electro #acidhouse http://t.co/FyRilIUnUx” | | | (Yes=1, No=0) |
| Hashtag | If a tweet text included on of the following suspicious hashtags, then this was coded “yes.”   - #Ebolafootballchants - #falsenews - #andnowihaveebola - #cdcwhistleblower - #tcot - #thewalkingdead - #p2 - #gossip - #UniteBlue - #the_walking_dead - #ebolazombie - #walkingdead - #betterebolaczars - #scarystoriesin5words - #zombie - #TwitterJoke - #EbolaJokes - #Ebolahoax - #Lies - #obola - #Ebolawatch - #fearbola - #Mandatoryquarantine | | | (Yes=1, No=0) |
| Code Name | Code Description and Example | Measure | Kappa Value | Raw Agreement |
| Tweet is a Joke | If tweet was perceived to be an obvious use of humor or sarcasm without aggression/anger or the intent to sow discord, this was marked “yes”.  Example: “Warning!!!! If you get an email claiming you can catch Ebola from eating tinned food. Don't worry it's just spam!!” | (Yes=1 , No=0) | 0.95 | 98.50% |
| Statement in Tweet True | If the tweet text contained information that was in line with substantiated facts given during the time the tweet was sent, then this was marked “yes”. If the tweet referenced information with unknown veracity, coders would search for reputable sources to support evidence and lean towards coding “Unable to ascertain the truth of statement in tweet”  Example: “Dallas officials: 120 people still being monitored for #Ebola: http://t.co/W7mWuZJRfu” | (Yes=1 , No=0) | 0.92 | 96.50% |
| Statement in Tweet Half-true, Misconstrues the truth | If the tweet text contained a statement that misinterpreted information around ebola (included things like misperceptions about testing vs cases in cities and over stating the results of modeling), then this was coded “yes”. If the tweet referenced information with unknown veracity, coders would search for reputable sources to support evidence and lean towards coding “Unable to ascertain the truth of statement in tweet”  Example: “ The phrase “expelled into the air means that there is clearly the existence of the “airborne transmission of Ebola”. #tcot sneezing, coughing” | (Yes=1 , No=0) | 0.95 | 99.50% |
| Statement in Tweet False | If the tweet text lists information that clearly differentiates from that of consensus expert opinion at the time of its publishing, then mark this “yes”. If the tweet referenced information with unknown veracity, coders would search for reputable sources to support evidence and lean towards coding “Unable to ascertain the truth of statement in tweet”  Example: “Weaponized Ebola Was Created By South Africa in 1980's: US Was Involved — And There's No Evidence It Was Destroyed http://t.co/V8CChPyAw5” | (Yes=1 , No=0) | 0.94 | 99.50% |
| Unable to Ascertain the Truth of Statement in Tweet | If the tweet text listed information where it was not feasible for coders to provide a definitive answer, then this code was marked “yes”  Example: “Ron Klain was not in Obama's meeting on Ebola response today” | (Yes=1 , No=0) | 0.83 | 97.00% |
| Statement in Tweet Meant to Share Opinion | If the tweet text contained a statement that was meant to share an opinion, then this code was marked “yes”  Example: “Ebola may be in the headlines, but tobacco is the real killer in Africa http://t.co/A1QAG7MD2o” | (Yes=1 , No=0) | 0.87 | 94.00% |
| Statement in Tweet Promotes Discord | This code was based off Broniatowski et al’s paper examining the weaponization of health communication.^x^ If the tweet text included jokes, snark, or sarcasm that was designed to agitate, demonstrate aggression, be pointed, bait users into responding, then this code was marked “yes”  Example: “Ebola is just an excuse for the government to control you without making it look like they're "trying" to control you. #OpenYourEyes” | (Yes=1 , No=0) | 0.85 | 95.00% |
| Political Context |  |  |  |  |
| Statement includes sentiments related to politics/government | If the tweet text referenced a government official or agency’s actions, then this code was marked “yes”  Example: @GovernorPerry At the Texas A&amp;M National Center for Therapeutics Manufacturing. #Ebola #Zmap #TXPREP | (Yes=1 , No=0) | 0.91 | 97.00% |
| Statement in tweet in support of government | If the tweet text referenced political officials or agencies in a positive light, then this code was marked “yes”  Example: “RETWEET if you agree with @TedCruz: we must close our borders to Ebola afflicted countries. http://t.co/uICS8iJodb” | (Yes=1 , No=0) | 0 | 99.50% |
| Statement in tweet opposed to government | If the tweet text referenced political officials or agencies in a negative light, then this code was marked “no”  Example: “Obama refusal to restrict travel to US from Ebola hot zones is a purely political ploy risking American lives. Hagan supports this. #NCSen” | (Yes=1 , No=0) | 0.90 | 98.00% |
| Risk Frame |  |  |  |  |
| Tweet includes risk increasing message | This category was based off of Sell et al’s 2017 article on message framing during the 2014 Ebola outbreak. If tweet text included themes that would increase risk perception in the audience, including messages regarding Ebola mortality, lack of countermeasures, inability to stop the outbreak, scientific misunderstanding of the virus, growth of the epidemic, then this code was marked “yes”  Example: “Rajani Ward of National Nurses United says 85% of nurses in country feel unprepared for Ebola.” | (Yes=1 , No=0) | 0.79 | 89.50% |
| Tweet includes risk minimizing message | This category was based off of Sell et al’s 2017 article on message framing during the 2014 Ebola outbreak. If tweet text included themes that would decrease risk perception in the audience, including messages regarding ability to stop transmission, low risk related to Ebola in the United States, scientific knowledge about ebola, then this code was marked “Yes”  Example: “ At this point in time we have NO evidence and do NOT anticipate that the #Ebola virus is mutating to become airborne” | (Yes=1 , No=0) | 0.82 | 99.00% |
| Ebola Specific Content |  |  |  |  |
| Tweet includes sentiments related to health | If the tweet text referenced ebola in the context of health, then this code was marked “yes”. This included statements regarding symptoms, testing, “patients”, disease outcomes, public health monitoring, and disease spread/recovery.  Example: "this Ebola strain appeared to carry a much higher “viral load” than previous strains." http://t.co/UBJ2hrjTvX” | (Yes=1 , No=0) | 0.83 | 99.00% |
| Tweet includes message about countermeasures, cure or vaccines | If the tweet text referenced cures, medical countermeasures, or cures, than this code was marked “yes”  Example: “My aunt's company in North Carolina is about to get the Ebola vaccine approved by the FDA and she's the project manager. Ur welcome errbody” | (Yes=1 , No=0) | 0.85 | 98.50% |
| Tweet includes message about fatal nature of Ebola | If the tweet text referenced the fatal nature of ebola, including statements about orphaned children, then this code was marked “yes”  Example: “Latest data shows Ebola deaths continue to follow an exponential model (source: http://t.co/mpczj4hBdL) http://t.co/qG9szQjG2n” | (Yes=1 , No=0) | 0.87 | 94.50% |
| Tweet includes message about spread of disease/growth of pandemic | If the tweet text referenced the growth of the Ebola epidemic, then this code was marked “yes”. This include messages about the growth of individual cases in new areas, but does not include statements around testing potential patients.  Example: “Here are all the people outside of Africa who have Ebola http://t.co/vHb6GMDfDh” | (Yes=1 , No=0) | 0.80 | 99.50% |
| Tweet includes message about the reduction of Ebola | If the tweet text referenced the reduction of cases or the epidemic spread, then this code was marked “yes”.  Example: “ "Happy! I’m so happy!" Adamsay, 11, who was declared #Ebola-free. Pic via @UNICEFSL http://t.co/KZ9ofEaNxc” | (Yes=1 , No=0) | 0.80 | 99.50% |
| Tweet includes message travel bans / closing the borders | If the tweet text referenced travel bans and closed borders within the context of the Ebola outbreak, then this code was marked “yes”.  Example: “Obama is making the Ebola problem much worse than it needs to be in the U.S. by not halting flights from West Africa. Airport testing a joke” | (Yes=1 , No=0) | 1.00 | 100.00% |
| Tweet includes message about quarantine or isolation | If the tweet text referenced quarantine or isolation at any level (individual, policy, structural), then this code was marked “yes”.  Example: “Spanish Ebola nurse reported symptoms many times before being quarantined http://t.co/a0INtwyob2” | (Yes=1 , No=0) | 1.00 | 100.00% |
| Tweet includes message about screening, passport or fever checks at border | If the tweet text referenced screening travelers, including passport and fever checks at the border, then this code was marked “yes”.  Example: ‘ Ebola screenings to begin at area airports, officials say \| Verizon FiOS1 News - New Jersey http://t.co/OLp26m52sf” | (Yes=1 , No=0) | 1.00 | 100.00% |
| Tweet includes message about public health monitoring | If the tweet referenced public health monitoring efforts, then this code was marked “yes”. This was exclusive to public health authority monitoring efforts and did not include messages about treating Ebola patients or Ebola testing.  Example: “ U.S. plans 21-day monitoring of people who arrive from Ebola-stricken nations http://t.co/Fri9zqVq5j” | (Yes=1 , No=0) | 0.80 | 99.50% |
| Rumors |  |  |  |  |
| Tweet mentions a rumor | If the tweet text referenced a rumor regarding ebola, then this code was marked “yes” and the corresponding rumor code was also marked “yes”. | (Yes=1 , No=0) | 0.93 | 99.00% |

| Drop Down List of Specific Rumors | Description and Example |
| --- | --- |
| Tweet refutes a rumor | If the tweet text directly refuted the rumor mentioned in the tweet, then this code was marked “yes”. This must be a direct refute of a rumor, and would not be coded if the tweet text presented information that would contradict a rumor without the context of the rumor.  Example: “The #Ebola virus is NOT spread through: casual contact, air, water, food grown or legally purchased in U.S. http://t.co/DaRfrXyykK” ***Refutes Rumor (Transmission)*** |
| Tweet mentions rumor about Ebola victims rising from the dead | If the tweet text referenced the rumor of Ebola patients rising from the dead, then this code was marked “yes”. This also included “ebola zombies”.  Example: “Ebola zombies?? think its time to go to the winchester and wait for all this to blow over” |
| Tweet mentions rumor about airborne transmission | If the tweet text referenced the rumor that Ebola virus is able to be spread via the air, then this code was marked “yes”.  Example: “ “@EmergencyHeaIth: Ebola may be airborne as of October 15th” *moves to canada*” |
| Tweet mentions rumor about Ebola in non-reported places | If the tweet text referenced Ebola in areas where it was unconfirmed, then this code was marked “yes”. This included spaces of varying sizes including US cities and less defined locations (see example below).  Example: “ If you're concerned about ebola, you probably shouldn't vote. High risk of contracting the disease at polling places.” |
| Tweet mentions rumor about contracting Ebola from common products | If the tweet text referenced common objects as a source of the Ebola virus, then this code was marked “yes”. This was driven from the Jin article that cited rumors of catching Ebola from objects like iPhones and hair extensions.  Example: “DEY SAY EBOLA HAS BEEN FOUND IN WEAVE .....U B*****S BETTA START GOIN NATURAL 😂😂😂💯👌💆💇” |
| Tweet mentions rumor about US involvement in the spread of Ebola | If the tweet text referenced a conspiracy/rumor that the United States was involved in the origin or increased impact of the Ebola outbreak, then this code was marked “yes”.  Example: “Understand the U.S. gov't origin of #Ebola &amp; #AIDS; Dr. Leonard Horowitz's groundbreaking book, Emerging Viruses http://t.co/yy8TvKXJnQ” |
| Tweet mentions rumor about safety of Ebola medical countermeasures | If the tweet text referenced questions about the safety of medical countermeasures used in the Ebola outbreak response, then this was marked “yes”. This was a rumor that was more prevalent in studies of West African social media.  Example: *This did not occur in our data set* |
| Tweet mentions rumor about foreigners bringing Ebola | If the tweet text referenced unsubstantiated rumors of foreigners bringing Ebola into the United States undetected, then this code was marked “yes”. This included the rumor that migrants from Central America were a potential source of Ebola entering the United States (see example below).  Example: “ GET READY: Marine Corps General Warns of 'Mass Migration Into U.S.' If Ebola Hits Central America http://t.co/lBlZlUqBqF via @Doug_Giles” |
| Tweet mentions rumor about new remedies or medical interventions | If the tweet text referenced new remedies, cures, or medical interventions for Ebola, then this code was marked “yes”.  Example: “ "Garcinia Kola" is the only cure for Ebola. Type it in on Instagram &amp; see what happens. Someone is trying to hide this info from the public.” |
| Tweet mentions rumor about the use of Ebola as a bioweapon | If the tweet text referenced the use of the Ebola as a bioweapon, or that the outbreak was the result of a bioweapon attack/release, then this code was marked “yes”.  Example: “is this mystery respiratory illness and spreading Ebola biological warfare? Pretty strange coincidence. http://t.co/LMDwciaIN5 #tcot” |
| Tweet mentions rumor about outbreak leading to fall of government | If the tweet text referenced that the Ebola outbreak would lead to the dissolution of the US government, then this code was marked “yes”. This included statements regarding turning to medical martial law.  Example: “If yall haven't did yall research on the Ebola , and fema , martial law and the new world order I highly recommend it. Go get educated.” |
| Tweet mentions rumor about Ebola being a product of divine intervention | If the tweet text referenced that the Ebola outbreak was the result of define intervention, then this code was marked “yes”. This also included statements that the virus was an act of religious vengeance.  Example: “ Audio: NC marriage activist warns of super Ebola that will destroy gay-accepting America http://t.co/cAiEA3wqjc” |
| Tweet mentions rumor about the Ebola outbreak being a hoax | If the tweet text referenced that the Ebola outbreak was actually fake or a hoax, then this code was marked “yes”.  Example: “Ebo-Lie: Man Living In Ghana Confirms Ebola Is A Hoax http://t.co/2yfrRMHZAs” |
| List other rumors not listed | If the tweet text referenced a rumor that was not included in our list, then this code was marked ”yes” and a brief text summary was entered.  Example: “Emory University backtracks: DeKalb Co. never threatened sewer cutoff over Ebola virus. <http://t.co/xC4VVfTkiz>” |
